# Modification of Docosahexaenoic Acid Composition of Milk from Nursing Women Who Received Alpha Linolenic Acid from Chia Oil during Gestation and Nursing

**DOI:** 10.3390/nu7085289

**Published:** 2015-08-04

**Authors:** Rodrigo Valenzuela, Karla A. Bascuñán, Rodrigo Chamorro, Cynthia Barrera, Jorge Sandoval, Claudia Puigrredon, Gloria Parraguez, Paula Orellana, Valeria Gonzalez, Alfonso Valenzuela

**Affiliations:** 1 Department of Nutrition, Faculty of Medicine, University of Chile, Av. Independencia 1027, Independencia, Santiago 8380453, Chile; E-Mails: kbascunan@med.uchile.cl (K.B.); rodrigochamorro@med.uchile.cl (R.C.); cynthia.barrera@gmail.com (C.B.); gparraguez@ug.uchile.cl (G.P.); p_orellana@ug.uchile.cl (P.O.); vale_elba@hotmail.com (V.G.); 2 Lipid Center, Institute of Nutrition and Food Technology (INTA), University of Chile, Av. El Líbano 5524, Macul, Santiago 8380453, Chile; E-Mail: avalenzu@inta.uchile.cl; 3 Obstetrics and Gynecology Department, Clinical Hospital of the University of Chile, Av. Santos Dumont 999, Independencia, Santiago 8380453, Chile; E-Mails: jsandoval@hcuch.cl (J.S.); cpuigrredon@hcuch.cl (C.P.)

**Keywords:** pregnancy and nursing, chia oil, erythrocyte phospholipids, ALA and DHA in milk

## Abstract

α-Linolenic acid (ALA) is the precursor of docosahexaenoic acid (DHA) in humans, which is fundamental for brain and visual function. Western diet provides low ALA and DHA, which is reflected in low DHA in maternal milk. Chia oil extracted from chia (*Salvia hispanica* L.), a plant native to some Latin American countries, is high in ALA (up to 60%) and thereby is an alternative to provide ALA with the aim to reduce DHA deficits. We evaluated the modification of the fatty acid profile of milk obtained from Chilean mothers who received chia oil during gestation and nursing. Forty healthy pregnant women (22–35 years old) tabulated for food consumption, were randomly separated into two groups: a control group with normal feeding (*n* = 21) and a chia group (*n* = 19), which received 16 mL chia oil daily from the third trimester of pregnancy until the first six months of nursing. The fatty acid profile of erythrocyte phospholipids, measured at six months of pregnancy, at time of delivery and at six months of nursing, and the fatty acid profile of the milk collected during the first six months of nursing were assessed by gas-chromatography. The chia group, compared to the control group, showed (*i*) a significant increase in ALA ingestion and a significant reduction of linoleic acid (LA) ingestion, no showing modification of arachidonic acid (AA), eicosapentaenoic acid (EPA) and DHA; (*ii*) a significant increase of erythrocyte ALA and EPA and a reduction of LA. AA and DHA were not modified; (*iii*) a increased milk content of ALA during the six months of nursing, whereas LA showed a decrease. AA and EPA were not modified, however DHA increased only during the first three months of nursing. Consumption of chia oil during the last trimester of pregnancy and the first three months of nursing transiently increases the milk content of DHA.

## 1. Introduction

Several studies have established the important physiological role of n-3 fatty acids in infant growth and development, especially their importance for neuronal and visual development [1,2]. n-3 Fatty acids are a family of essential nutrients derived from alpha linolenic acid (C18:3 n-3, ALA), the precursor of the physiologically active n-3 long-chain polyunsaturated fatty acids (n-3 LCPUFA), eicosapentaenoic acid (C20:5, EPA) and docosahexaenoic acid (C22:6, DHA) [3]. ALA is an essential nutrient for humans, and its deficiency produces neurological alterations in infants [4] and dermatological disorders in adults [5]. Through a series of enzymatic reactions of elongation and desaturation, which occurs mainly in the liver, ALA is first transformed into EPA and then to DHA, which is the main metabolic end product. DHA is involved in multiple functions in the human body where it exerts a central role in the physiological and normal development of the individual from the embryonic stage on [6]. Accretion of DHA is critical during pregnancy and during the first year of life in humans, because the fatty acid is essential for the formation and function of the nervous and visual systems [7]. DHA comprises 10% of the dry weight of the human brain, the fatty acid making up 35%–40% of the total brain LCPUFA [8]. In the nervous tissue, and particularly in the brain, DHA is critical to all aspects of neurodevelopment and brain function, including neurogenesis, neurite proliferation and growth, nerve impulse transmission via the sodium-potassium pump, neuronal integrity and vitality, blood glucose transport and gene expression in the brain [9,10,11]. The pregnant and nursing woman has a physiological requirement of n-3 LCPUFA, and specifically of DHA, to assure the adequate and normal growth and development of the child [12]. Transformation of ALA into its metabolic products (EPA and DHA) mainly occurs in the hepatic tissue through enzymatic processes of elongation and desaturation [13]. EPA is primarily directed to the formation of eicosanoid derivatives, which have anti-inflammatory actions and regulatory effects on endothelial vascular activity [14]. Almost all the DHA is transported to the placenta during pregnancy and actively accreted at the fetal brain and visual tissues [15]. After birth, DHA is provided to the newborn through the maternal milk, which contains a small but significant amount of DHA (0.30%–0.32%) [16]. The Western diet provides very low amounts of DHA, because it only comprises small amounts of the main suppliers of this fatty acid (marine foods) [17]. Therefore DHA supplementation for women during pregnancy and nursing has been suggested [18]. However, this supplementation is not easily accepted during the perinatal period because some nutritional supplements of DHA are derived from fish oil of which mothers may show low tolerance [19]. Because ALA is the nutritional precursor of DHA, it has been proposed that the ingestion of foods containing ALA may compensate the chronically low ingestion of DHA by woman during the perinatal period, providing that ALA be ingested in high enough amounts because of its low metabolic transformation to DHA (less than 1%) [20]. A wide variety of vegetable oils having a high content of ALA (30%–65%) are available at present, such as camelina oil (*Camelina sativa* L., 36%), perilla oil (*Perilla frutescens* L., 53%), chia oil (*Salvia hispanica* L., 60%–65%), flaxseed oil (*Linum usitatissimun* L., 57%), sacha inchi oil (*Plukenetia volubilis* L., 49%) [3]. These oils, which are produced and commercially available in many countries, particularly in South America, are possibilities for dietary supplementation of ALA with the aim of increasing the DHA levels in breast milk. Previous research of our group has demonstrated that supplying ALA from chia oil to adult rats results in increased accretion of DHA in several tissues, particularly in the liver and brain [21], suggesting an efficient transformation of ALA into DHA. Chia seed and its oil have been very well characterized in their chemical composition and antioxidant value [22]. In the present report we evaluated the effect of chia oil as the main daily source of ALA by measuring the DHA content of erythrocyte phospholipids and breast milk obtained from women who received the oil during a period of gestation and nursing.

## 2. Subjects and Methods

### 2.1. Study Design and Subjects

The study was a randomized clinical trial that included 40 pregnant women currently attended at the Obstetrical and Gynecology Health Service of the University of Chile Hospital. It was conducted during the period from January 2012 to December 2013. Inclusion criteria were: an age of 22–35 years, a gestational age of at least 22 to 25 weeks according to the date of the last menstrual period and confirmed by ultrasound, 1–4 children and a history of successful nursing. Recruited women mainly belonged to the low and middle socioeconomic status according to the European Society for Opinion and Marketing Research (ESOMAR) [23]. All were of Hispanic origin. Women with a history of drugs or alcohol consumption, a diet including polyunsaturated fatty acids (PUFA, ALA supplements) or LCPUFA (EPA and or DHA supplements), with underweight as defined by the Chilean chart for pregnant women [24], with a history of twins or of suffering from chronic diseases such as diabetes, arterial hypertension, obesity, or other illness that could affect fetal growth, were excluded from the study. At the time of recruitment, all women fulfilling the inclusion criteria were given general information about the study, and a dietitian explained the objectives and main characteristics of the study design. The study protocol was reviewed and approved by the Institutional Review Board of the Faculty of Medicine, University of Chile (Protocol #073-2011) and by the Ethics Committee of the Clinical Hospital, University of Chile (Protocol #507/11). All information regarding the study was given to each participant who voluntarily agreed to participate and signed the informed consent.

During the first appointment for nutritional evaluations, the pregnant women were randomly assigned to either the control group (*n* = 21) or to the experimental group that received the dietary supplementation with chia oil (chia group, *n* = 19). The fatty acid profile and ALA content (65%–68%) of the oil has previously been assayed by our group [21]. All the pregnant women received a complete nutritional interview including nutritional diagnosis and counseling according to the dietary guidelines for pregnant women. Both groups were counseled to have a controlled intake of vegetable oil (sunflower/soybean oil, 80:20 v/v) at home. Each woman was given plastic teaspoons (4 mL) that allowed measuring her consumption of vegetable oil. A previous study had demonstrated a good tolerance of pregnant women of chia oil [25]. The specific indication for the control group was to consume four teaspoons of uncooked vegetable oil per day (16 mL/day) mainly in salads at lunch and dinner. The Chia group was instructed to replace their intake of usual oil with chia oil (16 mL/day; 10.1 g ALA/day). Women belonging to each group were given 4500 mL of each oil in 250 mL bottles. The control and chia groups consumed the respective oils from the 6th month of pregnancy until the 6th month of nursing (total intervention: 9 months). All women received a dietary record to register the daily consumption of vegetable oil and were visited weekly to assess oil consumption. Chia oil obtained by cold pressing of chia seeds was a gift of Benexia Co. (Santiago, Chile).

### 2.2. Assessment of Nutritional Status

Participants were subject to a clinical evaluation when incorporated into the study. A physician and a nurse assessed each participant regarding health following the standard clinical approach for pregnant women. Anthropometric data of weight (kg) and height (m) were assessed to determine body-mass index (BMI, kg/m^2^). BMI was then used to establish maternal nutritional status according to gestational week following the Chilean reference [24]. Energy and nutrient requirements were established according to WHO criteria [26] and recommended dietary intakes according to the American Institute of Medicine, 2001 [27].

### 2.3. Dietary Intake of Mothers

All mothers were interviewed by a dietitian and asked to include all groups of consumed foods at the entry of the study, during the first week after delivery and six-months after delivery using a food frequency questionnaire. In addition to the food frequency questionnaire, dietitians used a photographic “Atlas of Commonly Consumed Foods in Chile” [28], a validated graphic instrument that helps to estimate the amount of each food consumed. Dietary data from the food-frequency questionnaire was grouped into nine food groups (cereals, fruits and vegetables, dairy, meats and eggs, legumes, fish and shellfish, high-lipid foods, oils and fats, sugars and processed foods). Cereals included all cereals and potatoes; fruits and vegetables included all kind of fruits, natural fruit juices and vegetables; dairy products included milk, cheese, fresh cheese and yogurts; meats and eggs included beef, chicken, pork and turkey meat, and all their derived products, and eggs; fish and shellfish included mackerel, tuna, salmon and shellfish (fresh and freeze); legumes included beans, chickpeas and lentils; high-lipid foods included olives, almonds, peanuts, walnuts, avocados, pistachios and hazelnuts; oils and fats included vegetable oils (mainly sunflower/soybean, canola, grape seed and olive oil) and fats (lard, butter, margarine, mayonnaise and cream); sugars and processes foods included sugar, honey, jam, delicacy, soft drinks, artificial juices, chocolates, cookies and sweet and savory snacks. Dietary data was analyzed using the software Food Processor SQL^®^ (ESHA Research, Salem, OR, USA), to calculate energy and nutrient intake. Diet composition was obtained using a database from the USDA National Nutrient Database for Standard Reference, which also contained information from locally generated nutrient composition data.

### 2.4. Collection of Blood and Breast Milk Samples

Blood samples were obtained at the entry of the trial, immediately after delivery and six months after delivery. Butylated hydroxytoluene (BHT) was added to the blood samples as antioxidant and the samples were immediately centrifuged to obtain the erythrocyte fraction (3000× g for 10 min at 20 °C) and then frozen at −80 °C until further analysis. Breast milk (5 mL) was extracted by the mothers themselves after the infant had been fed for at least 2 minutes and was collected in plastic vials. Milk samples were immediately frozen at −80 °C until further analysis. Frozen erythrocytes and milk samples were transported to the Biochemical Nutritional Laboratory—Lipid Research Area at the Department of Nutrition, Faculty of Medicine, University of Chile, for analytical procedures. 

### 2.5. Fatty Acid Analysis

#### 2.5.1. Lipids Extraction from Erythrocytes and Breast Milk

Quantitative extraction of total lipids from erythrocytes and breast milk was carried out according to Bligh and Dyer [29] with the addition of BHT. Erythrocytes and breast milk samples were separately mixed with ice-cold chloroform/methanol (2:1 v/v, containing 0.01% BHT), magnesium chloride was added (0.5 N), and the mixture was homogenized in an Ultraturrax homogenizer (Janke & Kunkel, Stufen, Germany). The total lipids extracted from erythrocytes and milk were separated by think layer chromatography (TLC) (aluminum sheets 20 × 20 cm, silica gel 60 F-254; Merck), using the solvent system hexane/diethylether/acetic acid (80:20:1 v/v). After the development of the plates and solvent evaporation, lipid spots were visualized by exposing the plates to a Camag UV (250 nm) lamp designed for TLC. The solvent system allows the separation of phospholipids, triacylglycerols, cholesterol and cholesterol esters according to their relative mobility. Spots corresponding to phospholipids were scraped from TLC plates and extracted by elution with either diethylether or chloroform/methanol (2:1 v/v), according to Ruiz-Gutierrez *et al.* [30]. 

#### 2.5.2. Preparation of Fatty Acid Methyl Esters (FAMEs)

Fatty acid methyl esters (FAMEs) from erythrocyte phospholipids and milk fatty acids were prepared according to Morrison and Smith [31]. Samples had previously been dissolved in chloroform/methanol (2:1 v/v) and were then evaporated under nitrogen stream until the volume was halved, then boron trifluoride (12% methanolic solution) and sodium hydroxide (0.5 N methanolic solution) were added and the mixture was cooled. FAMEs were extracted with 0.5 mL of hexane.

#### 2.5.3. Gas Chromatographic Analysis of FAMEs

FAMEs were identified and quantified by gas-liquid chromatography in an Agilent equipment (model 7890B, Santa Clara, CA, USA) equipped with a capillary column (Agilent HP-88, 100 m × 0.250 mm; I.D. 0.25 µm) and flame ionization detector (FID). The injector temperature was set at 250 °C and the FID temperature at 300 °C. The oven temperature at sample injection was initially set at 120 °C and was programmed to increase to 220 °C at a rate of 5 °C per min. Hydrogen was utilized as the carrier gas at a flow rate of 35 cm per second in the column, and the inlet split ratio was set at 20:1. Identification and quantification of FAMEs were achieved by comparing the retention times and the peak area% values of unknown samples to those of commercial lipid standard (Nu-Chek Prep Inc., Elysian, MN, USA). C23:0 was used as internal standard (Nu-Chek Prep Inc., Elysian, MN, USA) and data was processed using the Hewlett-Packard Chemstation software system.

### 2.6. Statistical Analysis

Dietary data were checked by contrasting the energy/nutrient intake data composition with dietary questionnaires, identifying potential outliers. In the case if outliers, a careful review of each food frequency questionnaire was done. A descriptive analysis was conducted, and the analysis of the variable’s distribution was done using a Shapiro–Wilk test. Results are expressed as the mean ± SD. Dietary nutrient intake and erythrocyte phospholipids and breast milk fatty acid composition at the three sampling points of the intervention were compared through one-way ANOVA and Newman-Keuls test. For all comparisons, statistical significance was set at α level ≤0.05. The statistical software used was SPSS v.15.0 (Chicago, IL, USA) and GraphPad Prism v. 6.0 (GraphPad Software, San Diego, CA, USA) for figure processing.

## 3. Results

### 3.1. Background and Anthropometric Data of Groups

Table 1 shows the background and anthropometric data of the total sample and of each group. The total sample was composed of young women (28.6 ± 5.8 years), mainly of middle socioeconomic status (70.9%), and having very similar gestational periods, gender birth weight and height of their children. No significant differences were observed for the chia group when compared the control group for all background and anthropometric data. 

**Table 1:** Background characteristics of both experimental groups.

Background characteristic	Group		
	Whole sample (*n* = 40)	Control (*n* = 21)	Chia (*n* = 19)
Age (mother), years ^a^	28.6 ± 5.8	28.3 ± 6.7	29 ± 4.7
Pre-pregnancy weight, kg ^a^	65.2 ± 11	65.9 ± 9.9	64.4 ± 12.4
Pre-pregnancy BMI, kg/m^2^ ^a^	24.9 ± 4.2	24.8 ± 3.7	24.9 ± 4.8
SES *			
High, %	13.9	19.0	5.3
Medium, %	70.9	66.7	73.7
Low, %	15.2	14.3	21.1
Gestational age at birth, weeks	38.6 ± 1.1	38.6 ± 1.1	38.7 ± 1.2
Gender, masc %	53.3	53.8	52.6
Birth weight, g	4065.2 ± 481.9	4013.2 ± 587.9	4136.5 ± 279.8
Birth height, cm	48.6 ± 3.5	49.1 ± 3.4	48.0 ± 3.6

Data are expressed as mean ± S.D., or percentage (%) when indicated. * SES: socioeconomic status assessed by using the ESOMAR criteria [22]; BMI: body mass index = kg/m^2^. ^a^ Anthropometric measures taken at the study enrollment.

### 3.2. Dietary Intake

The dietary intake of both groups is shown in Table 2. The energy and the macronutrient intake (carbohydrate, protein and fat), including fiber, of both groups was similar, with no significant differences. The exceptions were energy and carbohydrate consumption for the control group at the start (6th month of pregnancy) and at the end of the study (6th month of nursing). Total saturated fatty acid (SFA), monounsaturated fatty acid (MUFA) and polyunsaturated fatty acid (PUFA) ingestion were also similar in both experimental groups. However, as a result of the intervention, significant differences were observed for n-6, n-3 PUFA and some individual fatty acids (LA and ALA). At the point of delivery and at 6th month of nursing, compared to 6th month of pregnancy (start of intervention) a significant increase of ALA and a significant reduction of LA in the chia group was observed as expected from the chia oil intake. n-6/n-3 ratios were also significantly modified by chia oil ingestion. n-6 LCPUFA (AA) and n-3 LCPUFA (EPA and DHA) ingestion was not modified during the intervention. 

### 3.3. Fatty Acid Profile of Erythrocyte Phospholipids

Table 3 shows the fatty acid composition of erythrocyte phospholipids obtained from woman during pregnancy and the nursing period. The first sampling (6th month of pregnancy) showed no differences in the fatty acid profiles when control and chia groups were compared. However, at the second sampling (at delivery) some significant differences were observed. Although total SFA, total MUFA and total PUFA showed no modifications, differences were observed when total n-6 and total n-3 PUFA and some individual fatty acids were compared. The chia group, compared to the control group, showed a significant reduction in total n-6 PUFA, with LA and AA not being modified. Total n-3 PUFA, ALA and EPA were increased in the chia group, but DHA was not modified. The n-6/n-3 PUFA ratio was significantly reduced in the chia group. The third sampling (6 months of nursing) showed similar levels for total and individual fatty acids and for the n-6/n-3 ratio, as was observed for the second sampling (at delivery). 

**Table 2:** Energy and composition of diet ingested by mothers during pregnancy and nursing.

	6th Month of Pregnancy		Delivery		6th Month of Nursing	
Energy/Nutrients	Control group (a)	Chia group (b)	Control group (c)	Chia group (d)	Control group (e)	Chia group (f)
Energy (kcal)	2909 ± 426 ^(e)^	2057 ± 642.8	2477 ± 764.4	2119 ± 444.1	2287 ± 593 ^(a)^	1832 ± 510
Protein (g)	110.2 ± 30.1	94.6 ± 73	103.4 ± 30.1	81.7 ± 22.9	88.5 ± 37	74.2 ± 22.2
Carbohydrate (g)	400.7 ± 83.7 ^(e)^	275.7 ± 108.2	330.9 ± 154.9	276.1 ± 78.4	283.5 ± 72.9 ^(a)^	207.8 ± 54.2
Fat (g)	102.8 ± 30.5	85.0 ± 40.7	87.3 ± 24.9	66.8 ± 24.1	93.9 ± 31.4	66.1 ± 26.2
Cholesterol (mg)	312.2 ± 78.8	281.2 ± 190.2	328.4 ± 129.8	226.4 ± 79.9	277.4 ± 135.7	227.0 ± 61.6
Trans fatty acid (g)	2.2 ± 3.0	1.0 ± 0.7	1.3 ± 0.7	1.6 ± 1.1	1.5 ± 1.1	1.6 ± 0.9
Fiber (g)	35.4 ± 8.5	24.3 ± 10.4	25.9 ± 10.6	22.3 ± 7.6	24.8 ± 9.9	18.6 ± 8.2
SFA (g)	31.4 ± 11.0	24.7 ± 12.6	28.2 ± 11.2	24.9 ± 6.6	27.4 ± 11.5	20.6 ± 6.6
MUFA (g)	26.8 ± 11.0	22.9 ± 12.7	22.9 ± 8.2	24.6 ± 5.8	27.3 ± 14.5	23.8 ± 4.6
PUFA (g)	17.2 ± 5.2	19.3 ± 3.3	17.6 ± 3.9	21.5 ± 2.5	18.5 ± 2.7	17.9 ± 3.8
n-6 PUFA	15.4 ± 1.2 ^(d,f)^	17.5 ± 2.3 ^(d,f)^	16.3 ± 2.7 ^(d,f)^	11.5 ± 2.1 ^(a,b,c,e)^	16.9 ± 1.7 ^(d,f)^	10.1 ± 1.3 ^(a,b,c,e)^
n-3 PUFA	1.7 ± 0.05 ^(d,f)^	1.6 ± 0.04 ^(d,f)^	1.3 ± 0.2 ^(d,f)^	10.0 ± 1.4 ^(a,b,c,f)^	1.5 ± 0.04 ^(d,f)^	7.8 ± 0.9 ^(a,b,c,e)^
18:2, n-6 (LA) (g)	15.2 ± 3.0 ^(d,f)^	17.3 ± 3.3 ^(d,f)^	16.1 ± 2.6 ^(d,f)^	10.9 ± 2.3 ^(a,b,c,e)^	16.7 ± 3.3 ^(d,f)^	9.8 ± 1.8 ^(a,b,c,e)^
18:3, n-3 (ALA) (g)	1.1 ± 0.5 ^(d,f)^	1.2 ± 0.6 ^(d,f)^	1.1 ± 1.0 ^(d,f)^	9.5 ± 4.9 ^(a,b,c,e)^	0.9 ± 0.7 ^(d,e)^	7.7 ± 4.3 ^(a,b,c,e)^
20:4, n-6 (AA) (g)	0.08 ± 0.06	0.06 ± 0.06	0.09 ± 0.06	0.05 ± 0.02	0.08 ± 0.06	0.05 ± 0.02
20:5, n-3 (EPA) (g)	0.05 ± 0.07	0.03 ± 0.02	0.04 ± 0.05	0.03 ± 0.01	0.04 ± 0.02	0.005 ± 0.01
22:6, n-3 (DHA) (g)	0.1 ± 0.1	0.04 ± 0.05	0.07 ± 0.07	0.03 ± 0.03	0.04 ± 0.04	0.02 ± 0.03
n-6/n-3 PUFA ratio	9.1 ± 2.5 ^(d,f)^	10.9 ± 2.3 ^(d,f)^	12.5 ± 2.4 ^(d,f)^	1.15 ± 4.0 ^(a,b,c,e)^	11.3 ± 2.4 ^(d,f)^	1.30 ± 0.3 ^(a,b,c,e)^

Data are expressed as the mean ± SD for *n* = 21 women (Control group) and *n* = 19 (Chia group). Statistical significance (*p* < 0.05), ^a^: significantly different from Control group at 6th month of pregnancy; ^b^: significantly different from Chia group at 6th month of pregnancy; ^c^: significantly different from Control group at delivery; ^d^: significantly different from Chia group at delivery; ^e^: significantly different from Control group at 6th month of nursing; ^f^: significantly different from Chia group at 6th month. One-way ANOVA and Newman-Keuls test. Saturated fatty acids (SFA). Monounsaturated fatty acids (MUFA) Polyunsaturated fatty acids (PUFA).

**Table 3:** Fatty acid composition of erythrocyte phospholipids of mothers during pregnancy and nursing.

	6th Month Pregnancy		Delivery		6th Month Nursing	
Fatty acids (g/100 g of FAME)	Control group (a)	Chia group (b)	Control group (c)	Chia group (d)	Control group (e)	Chia group (f)
Total SFA	52.3 ± 4.5	53.6 ± 4.7	53.6 ± 3.3	50.2 ± 5.1	50.6 ± 4.1	49.7 ± 3.8
Total MUFA	12.3 ± 1.2	13.5 ± 0.9	11.7 ± 0.8	13.4 ± 1.1	15.9 ± 1.1	14.5 ± 0.9
Total PUFA	35.4 ± 3.0	32.9 ± 3.6	34.7 ± 2.9	36.4 ± 3.2	33.5 ± 3.3	35.8 ± 2.9
Total n-6 PUFA	28.7 ± 2.2	26.1 ± 3.3	27.1 ± 2.7	21.6 ± 1.8 ^(a,b,c,e)^	27.2 ± 1.4	20.2 ± 1.4 ^(a,b,c,e)^
Total n-3 PUFA	6.70 ± 0.8	6.80 ± 0.2	7.60 ± 0.9	14.8 ± 1.7 ^(a,b,c,e)^	6.30 ± 1.1	15.6 ± 1.6 ^(a,b,c,e)^
18:2, n-6 (LA)	13.4 ± 1.3	12.6 ± 1.5	12.1 ± 1.1	9.11 ± 1.4 ^(a,b,c,e)^	12.8 ± 1.6	8.02 ± 1.3 ^(a,b,c,e)^
18:3, n-3 (ALA)	1.03 ± 0.3	0.96 ± 0.2	1.02 ± 0.3	6.12 ± 2.3 ^(a,b,c,e)^	0.94 ± 0.2	7.39 ± 1.3 ^(a,b,c,e)^
20:4, n-6 (AA)	14.1 ± 1.6	13.2 ± 1.4	13.9 ± 1.2	12.2 ± 1.4	13.8 ± 1.4	11.9 ± 1.7
20:5, n-3 (EPA)	0.91 ± 0.1	0.89 ± 0.1	0.97 ± 0.3	2.58 ± 0.7 ^(a,b,c,e)^	0.86 ± 0.2	2.13 ± 0.8 ^(a,b,c,e)^
22:6, n-3 (DHA)	4.52 ± 0.8	4.68 ± 0.6	4.98 ± 1.0	5.33 ± 1.3	4.42 ± 1.1	5.10 ± 0.7
n-6/n-3 PUFA ratio	4.28 ± 0.9	3.83 ± 0.7	3.57 ± 0.7	1.46 ± 0.4 ^(a,b,c,e)^	4.31 ± 1.0	1.30 ± 0.3 ^(a,b,c,e)^

Data are expressed as g fatty acid per 100 g fatty acid methyl esters (FAME) and represent the mean ± SD for *n* = 21 women (Control group) and *n* = 19 (Chia group). Statistical significance (*p* < 0.05), ^a^: significantly different from Control group at 6th month of pregnancy; ^b^: significantly different from Chia group at 6th month of pregnancy; ^c^: significantly different from Control group at delivery; ^d^: significantly different from Chia group at delivery; ^e^: significantly different from Control group at 6th month of nursing; ^f^: significantly different from Chia group at 6th month. One-way ANOVA and Newman-Keuls test. Saturated fatty acids (SFA) correspond to 6:0, 8:0, 10:0, 12:0, 14:0, 16:0, 18:0, 20:0 and 22:0, 24:0. Monounsaturated fatty acids (MUFA) correspond to 14:1 n-5, 16:1 n-7 and 18:1, n-9. Polyunsaturated fatty acids (PUFA) correspond to 18:2 n-6, 18:3,n-3, 20:4 n-6, 20:5 n-3, 22:5 n-3 and 22:6 n-3; n-6/n-3 ratio is 20:4 n-6/ (20:5, n-3 + 22:5, n-3 + 22:6, n-3).

### 3.4. Fatty Acid Profile of Breast Milk

**Figure 1:**
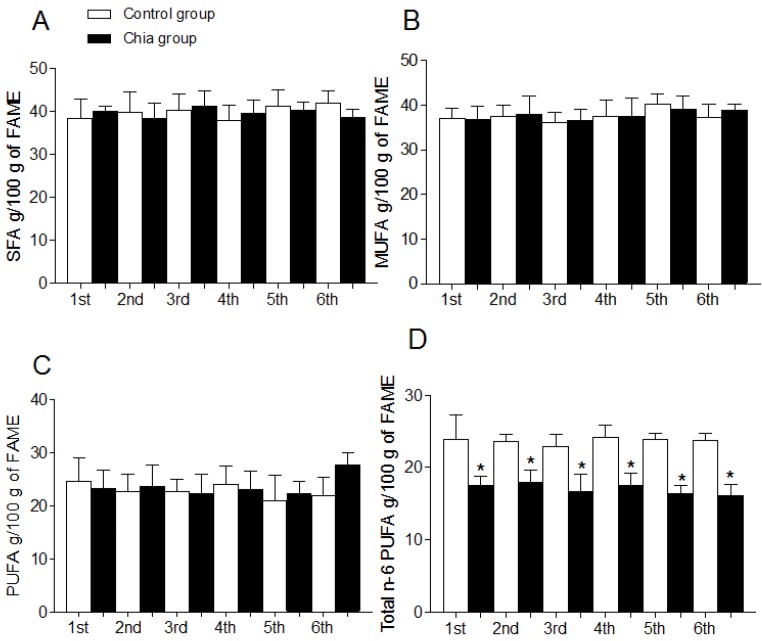

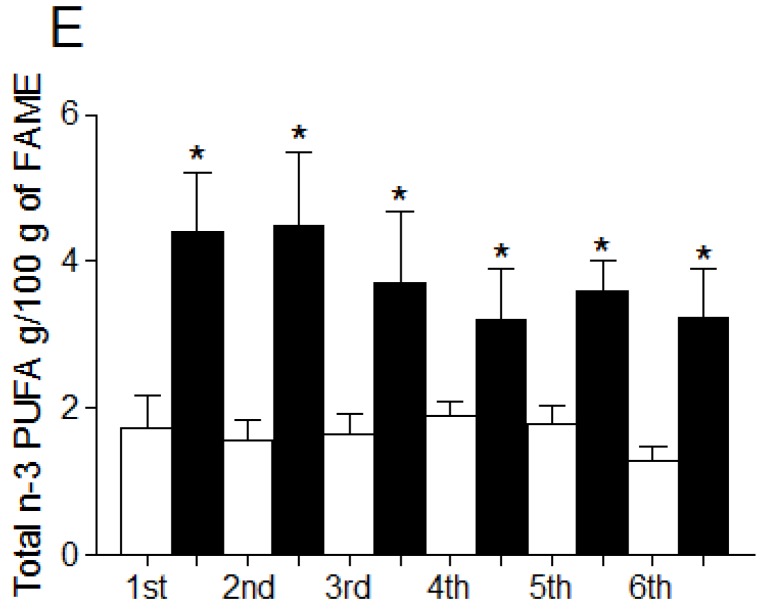
Total fatty acid composition of breast milk from mothers during nursing. Saturated fatty acids (SFA) (**A**); Monounsaturated fatty acids (MUFA) (**B**); Polyunsaturated fatty acids (PUFA) (**C**); Total n-6 PUFA (**D**); Total n-3 PUFA (E). Data are expressed as g fatty acid per 100 g FAME and represent the mean ± SD for *n* = 21 women (control group) and *n* = 19 (chia group). Statistical significance (*p* < 0.05); *: indicates significantly different when comparing the chia group with the control group for each month of nursing (*t*-test) and for all months of nursing (One-way ANOVA and Newman-Keuls test). SFA correspond to 6:0, 8:0, 10:0, 12:0, 14:0, 16:0, 18:0, 20:0 and 22:0, 24:0. MUFA correspond to 14:1 n-5, 16:1 n-7 and 18:1, n-9. PUFA correspond to 18:2 n-6, 18:3 n-3, 20:4 n-6, 20:5 n-3, 22:5 n-3 and 22:6 n-3.

**Figure 2:**
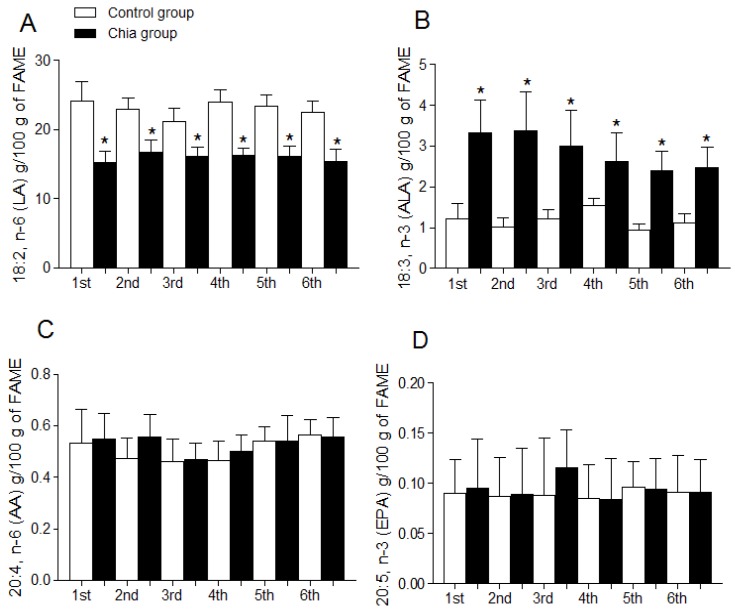

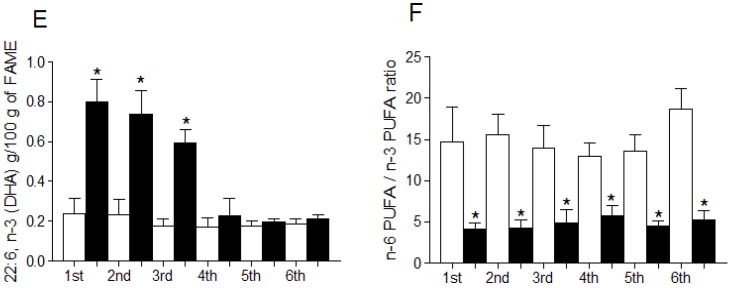
Individual fatty acid composition of breast milk from mothers during nursing. 18:2, n-6 (LA) (**A**); 18:3, n-3 (ALA) (**B**); 20:4, n-6 (AA) (**C**); 20:5, n-3 (EPA) (**D**); 22:6, n-3 (DHA) (E); (F). Data are expressed as g fatty acid per 100 g FAME and represent the mean ± SD for *n* = 21 women (control group) and *n* = 19 (chia group). Statistical significance (*p* < 0.05); *: indicates significant difference when comparing the chia group with the control group for each month of nursing (*t*-test) and for all months of nursing (One-way ANOVA and Newman-Keuls test). n-6 PUFA/n-3 PUFA ratio is (18:2, n-6 + 20:4, n-6)/(18:3, n-3 + 20:5, n-3 + 22:5, n-3 + 22:6, n-3).

Total SFA, MUFA, PUFA, and total n-6 PUFA and n-3 PUFA of breast milk are shown in Figure 1A–E. Total SFA (Figure 1A), total MUFA (Figure 1B) and total PUFA (Figure 1C) were not modified during the dietary intervention with chia oil when compared to the control group. Total n-6 PUFA (Figure 1D) were significantly reduced and total n-3 PUFA (Figure 1E) were significantly increased after chia oil intake. Figure 2 shows the individual modification of the most relevant n-6 and n-3 fatty acids and the n-6/n-3 PUFA ratio after chia oil intake. LA was significantly reduced in the chia group (Figure 2A) whereas ALA was significantly increased (Figure 2B) during all the periods of chia oil intake. AA (Figure 2C) and EPA (Figure 2D) were not modified in these groups. However, DHA (Figure 2E) was significantly increased in the chia group only during the first, second and third month of nursing, returning to values similar to the control group after the initial three-month period. The n-6/n-3 PUFA ratio (Figure 2F) was significantly reduced in the chia group during the six months of nursing.

## 4. Discussion

Dietary fatty acid intake by pregnant and nursing women in the supplementation periods studied is reflected in the fatty acid composition of erythrocyte phospholipids and also in the fatty acid composition of breast milk. Differences in the composition of fatty acids are particularly relevant for ALA, the precursor of n-3 LCPUFA, which in turn reflects the complex metabolism of polyunsaturated fatty acids and their conversion (through elongation and desaturation) to fatty acids of 20 and more carbon atoms [3,32]. In the same direction, our results demonstrate that ALA provided to pregnant and nursing women through chia oil increases (i) ALA and EPA content of erythrocyte phospholipids and (ii) ALA and DHA content of breast milk, but iii) does not modify DHA in erythrocyte phospholipids, which reflects that ALA conversion to n-3 LCPUFA and posterior accretion to cells or biological fluids, such as milk, is a highly regulated process [33]. Results for erythrocytes obtained from the chia group are in line with earlier observations by Arterburn *et al.*, (2006), who suggest that these cells are not metabolic reservoirs for DHA, as opposed to other tissues, such as brain cortex, sperm and retina [34]. However, erythrocytes are considered a good blood marker of the nutritional status of fatty acids [35]. Lauritzen and Carlson [36] have proposed that the maternal erythrocyte fatty acid profile during pregnancy and nursing is related to the fatty acid profile of the newborn’s erythrocytes, which indicates an active role of the placenta during pregnancy and of breast milk during nursing [37]. It has been previously demonstrated that dietary ALA intake increases EPA accretion in erythrocytes [38]. However, it has been also demonstrated that ALA intake, supplied as sacha inchi oil (49% ALA), increases both fatty acids, EPA and DHA, in erythrocytes [39,40], thus introducing controversy regarding the metabolism of n-3 PUFA in humans. 

During the six months of maternal milk analysis it was observed that daily intake of chia oil allowed a higher and more constant content of ALA. Similarly, an equal behavior for DHA was expected, as it is the product of the supposed continued transformation of ALA in the liver [41,42]. Both fatty acids are transported to the breast to be secreted in the milk [43]. Surprisingly, the increase of DHA in milk was only observed during the first, second and third months of nursing, reaching values similar to those of the control group after the third month of nursing in spite of the high and continuous ingestion of ALA. EPA did not increase either, as it has been shown to occur in populations were EPA ingestion via marine foods is high [34,44]. These observations lead to the hypothesis of a high control of the conversion of ALA to EPA and DHA, and of the presence of these fatty acids in breast milk. Breast milk does not contain EPA because this fatty acid competes with AA [45]. This is the reason why early formulas were enriched with DHA and AA from egg phospholipids excluding EPA [46]. We hypothesize that the physiological control of the conversion of ALA to DHA is produced: (i) through a high regulation in the activity of the enzymes involved in elongation and desaturation, which set DHA at physiological concentrations [47]; (ii) through the beta oxidation of exceeding ALA for energy production or carbon recycling [48]; (iii) due to the fact that excess of PUFA and LCPUFA may increase milk susceptibility to oxidative rancidity with the risk of tissue oxidative stress in the child, however this last proposal requires further demonstration. 

Literature indicates that women’s breast milk DHA values may vary from 0.2% to 1% of total fatty acids [34,49]. These values are highly dependent on the direct ingestion of DHA (*i.e.*, eating fish and/or taking n-3 LCPUFA supplements) as has been previously demonstrated [50]. In a recent study it was established that Chilean pregnant women who consistently show very low consumption of fish and other marine foods [51], as a result show a very low level of DHA in their breast milk as well as a low content of DHA in erythrocyte phospholipid fatty acids [51]. Populations that consume high amounts of fish, such as Philippine and Japanese women [52,53], show higher levels of DHA in milk compared to populations that consume less fish (e.g., Israel, Columbus, Ohio, USA) [54,55]. However the EPA content of the milk of women from these countries is very low and is not modified by fish consumption despite the presence of this fatty acid in marine food [34], indicating a physiological control in the transport and accretion of this fatty acid in the fat content of breast milk.

According to our results, ALA ingestion during the perinatal period only allows an increase of milk DHA during the first three months of nursing, suggesting either a limited further conversion and accretion of DHA in the liver and/or a regulated transport to the breast, as was postulated above. After the first three-month period of nursing, milk DHA reaches the estimated physiological levels for DHA (on average 0.3% to 0.32% of total fat) [56,57] despite the high nutritional availability of ALA. It is interesting that the content of AA and EPA of breast milk was not modified by ALA supplementation in our experimental model. This is highly relevant because of the close relationship of these two fatty acids with the control of vascular homeostasis and inflammatory responses in the infants [58]. In our study, AA was always present in erythrocytes of both experimental groups in the estimated physiological concentrations corroborating the important role of this fatty acid, such as for the brain development at early stages of life, as has been previously demonstrated [59].

Mothers that consumed chia oil were indicated to replace the habitual dietary vegetable oils (most commonly sunflower/soybean oil). This substitution which increases ALA consumption, allowed the replacement of a high proportion of LA for ALA, both in erythrocytes and in milk. It has been demonstrated that mothers that had a high dietary intake of LA and a low intake of ALA delivered children with subnormal scores of learning, where the high intake of LA was correlated with lower levels of DHA in breast milk [60]. A relevant result of the present study was the change in the total ratio of n-6/n-3 fatty acids in erythrocyte phospholipids and maternal milk. Ancestral modifications of this ratio in favor of n-3 fatty acids may have established a generic—evolutionary pattern, which at present characterizes the brain of humans [61].

Our results were obtained from non-obese mothers free of any chronic diseases, both aspects which are relevant because in obese women, mostly those suffering of nonalcoholic fatty liver disease, there is a reduction in the hepatic activity of desaturase enzymes (Δ-5 and Δ-6 desaturases) with a concomitant reduction in the formation of EPA and DHA from ALA [62]. Due the high prevalence of women obesity in western countries and comorbid nonalcoholic fatty liver disease [63], it is an interesting challenge for the future to evaluate the effect of ALA supplementation through chia oil and the presence of this fatty acid and of DHA in the milk secretion of these women. Our study did not evaluate the effect that the dietary intervention of pregnant and nursing women with chia oil had on their babies, such as length of gestational period, birth weight and cognitive and visual development. Other reports have studied the impact of DHA supplementation on these parameters [64,65], but not of ALA in concentrations as those used in our study. A previous study supplied canola oil (10% ALA) to pregnant women, showing an increase of the gestational period and birth weight [66]. 

## 5. Conclusions

Chia oil may constitute an available and inexpensive way to provide ALA in higher amounts to the population of many countries characterized by low fish consumption [51,67]. It is not of minor importance considering the actual low availability of fish and the increasing concerns about fish contamination with heavy metals and other toxic products that negatively influence fish consumption [68,69]. Our research has demonstrated that chia oil intake, a natural good source of ALA, allows an important modification in the EPA content of erythrocytes in pregnant mothers and an interesting increase of DHA in their milk. However, more research is necessary related to pre- and postnatal nutritional interventions with chia oil or other oils with a high content of ALA to scientifically support the recommendation of ALA consumption to increase the DHA content of breast milk. Chia oil supplementation may also contribute to improve the LA/ALA ratio in women during the perinatal period. 
